# Establishment and characterization of adult human gastric epithelial progenitor‐like cell lines

**DOI:** 10.1111/cpr.13355

**Published:** 2022-11-04

**Authors:** Yuan Gao, Ji Dong, Shuyue Qi, Xin Zhou, Xinglong Wu, Wendong Wang, Lu Wen, Wei Fu, Fuchou Tang

**Affiliations:** ^1^ School of Life Sciences, Biomedical Pioneering Innovation Center, Department of General Surgery Third Hospital, Peking University Beijing China; ^2^ Peking‐Tsinghua Center for Life Sciences, Academy for Advanced Interdisciplinary Studies Peking University Beijing China; ^3^ Beijing Advanced Innovation Center for Genomics Ministry of Education Key Laboratory of Cell Proliferation and Differentiation Beijing China; ^4^ Guangzhou Laboratory Guangzhou China; ^5^ Peking University Third Hospital Cancer Center Peking University Third Hospital Beijing China; ^6^ College of Animal Science and Technology Hebei Agricultural University Baoding Hebei China

## Abstract

**Objective:**

Human gastric epithelial stem/progenitor cells are important for stomach homeostasis; however, the in vitro culture system of these cells remains immature. Although three‐dimensional (3D) organoid culture has fundamentally changed the in vitro study of gastrointestinal tract, its use is limited by inaccessible luminal compartment, and difficulties of imaging and manipulation. To overcome these limitations of 3D organoid culture system, we established adult human gastric epithelial progenitor‐like (hGEPL) cell lines using a novel robust monolayer cell culture system.

**Materials and Methods:**

We established an in vitro gel‐based monolayer culture system for normal human adult gastric epithelium, and compared it with traditional two‐dimensional (2D) and 3D organoid culture systems using transcriptomics, immunofluorescence and cell viability experiments. At the same time, we used single‐cell transcriptomics to compare the differences of the hGEPL cells in conditioned medium (Cond.) and in chemically defined medium (Chem.), the two most common media for organoid culture, in maintaining the stemness and proliferative activity of hGEPL cells. Finally, we explored the role of key niche factors in inducing hGEPL cell differentiation.

**Results:**

The hGEPL cells were similar to the in vivo gastric epithelial stem/progenitor cells, which could stably proliferate in culture for a long time. Based on the established culture system, we explored signalling pathways that were important for the homeostasis of hGEPL cells. We found that after blocking the WNT signalling pathway or activating the BMP signalling pathway, hGEPL cells could differentiate into mucous surface cells.

**Conclusion:**

Our culture system of hGEPL cells from adults is robust and easy to operate, and has the transformative potential of personalized and precision medicine, laying a solid foundation for studying the self‐renewal and differentiation potentials of gastric epithelial stem/progenitor cells as well as modelling of related gastric diseases.

## Graphical abstract

We established a stable 2D monolayer culture system for gastric epithelial cells in adults and explored their potential for self‐renewal and differentiation. The cells we cultured had the expression of gastric marker genes and, by transcriptome analysis, were similar to gastric stem/progenitor cell types in vivo. We found that after blocking the WNT signalling pathway or activating the BMP signalling pathway, cultured cells could differentiate into mucous surface cells.

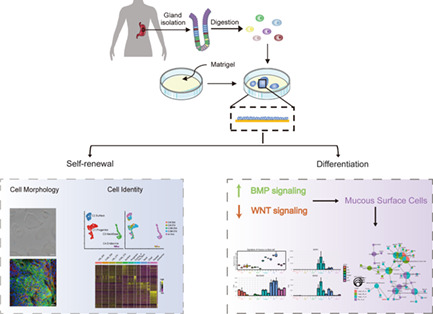

## 1 INTRODUCTION

Located between the oesophagus and the duodenum, human stomach is an important organ for storing, mixing and digesting food.^1^ Gastric mucosa is composed of thousands of gastric units with a wide variety of well‐organized cell types on the surface and in the gastric glands. The mucous surface cells secrete a large amount of alkaline mucous to protect the gastric mucosa from damage. Gastric glands contain a variety of secretory cells, such as parietal cells, neck mucous cells, chief cells and endocrine cells.^2^

Gastric stem cells (GSCs) are adult stem cells existing in the gastric tissue, which can not only maintain self‐renewal, but also differentiate into various gastric cell types under appropriate conditions. The stomach epithelium is capable of rapid renewal: the pit region has a rapid turnover rate of roughly 3–5 days, and the renewal rate will be faster if it is stimulated by certain external stimuli.^3^ For the gastrointestinal tract, which is vulnerable to external stimuli, the gastrointestinal epithelium may undergo multiple rounds of damage and repair, often in response to excessive inflammations. Inflammatory reaction is a double‐edged sword for stem cells. On the one hand, the gastrointestinal tract is the main source of reactive oxygen species (ROS). When the epithelial layer is damaged, ROS will activate immune cells, cause a series of immune reactions and damage GSCs, thus leading to a series of gastrointestinal diseases such as gastroduodenal ulcer and inflammatory bowel disease.^4^ On the other hand, studies have shown that the pro‐inflammatory environment induced by hyperbaric oxygen can enhance the differentiation of human mesenchymal stem cells to an osteogenic phenotype, which helps to enhance the differentiation ability of stem cells.^5^ Therefore, GSCs are important for maintaining normal physiological functions of the stomach, and the development of methods for in vitro culture of GSCs is critical for a better understanding of gastric homeostasis and stem cell biology.

Three‐dimensional (3D) organoid culture, which forms an expanding, self‐organizing epithelial structure with various cell types, is the most used in vitro culture system of gastric epithelium.^6^, ^7^, ^8^ 3D organoid cultures often have multiple cell types and are heterogeneous, which is a significant feature. 2D culture systems have the characteristics of easier genetic manipulation and imaging. Besides the traditional 2D culture system (direct culture of cells on a dish without coating), many novel 2D culture systems have been established, which have higher scalability and homogeneity. There are two common methods for novel 2D cell culture. One is to culture cells on the surface of the extracellular matrix coating, which generates several different culture systems in the small intestine and colon of mice to support the rapid expansion of their epithelial cells.^9^, ^10^, ^11^, ^12^ The other is based on the transwell system, a combination of epithelial monolayer culture on the transwell insert and fibroblast or immune cell culture on the transwell plate.^13^ However, these culture systems have some limitations, such as relatively poor long‐term maintenance during culture and difficulty of manipulation.

In this study, we attempted to expand human adult gastric epithelial cells on a large scale in vitro based on 2D monolayer culture system. First, we screened and optimized the culture system to make primary human gastric epithelial cells proliferate steadily for a long time in culture. Second, we systematically evaluated the cultured cells based on the morphological and transcriptomic analyses, and found that their gene expression patterns were very similar to those of in vivo gastric stem/progenitor cells. Third, using this system, we further explored the signalling pathways important for the self‐renewal and differentiation of these cultured hGEPL cells. In summary, we established a monolayer culture system for human adult gastric epithelial progenitor‐like cells, which was robust, easy to operate and optimal for long‐term culture. Our study lays a foundation for the understanding of critical biological features of human gastric epithelial stem/progenitor cells.

## 2 MATERIALS AND METHODS

### 2.1 Human gastric glands isolation

This study was approved by the Ethics Committee of Peking University Third Hospital (Licence no. IRB00006761‐M2016170), and all patients signed written informed consent for this study. Human gastric tissues were derived from clinical surgery and were rinsed with wash buffer (100 units/ml penicillin and 100 μg/ml streptomycin [15140‐122, Gibco], and 100 μg/ml primocin [#anti‐pm, InvivoGen] in Dulbecco's phosphate‐buffered saline [DPBS; D8537, Sigma‐Aldrich]) two times. We isolated gastric glands based on the previously published protocol with some modifications.^7^ Briefly, blood vessels, adipose tissue, and mucus were carefully removed with forceps and scissors. And the tissue was washed with wash buffer again till the supernatant was clear. We cut the tissue into pieces, collected the pieces into a 50 ml centrifuge tube, and immersed them in 20 ml cold chelating buffer (10 mM ethylene diamine tetraacetic acid (EDTA; AM9261, Ambion) and 0.5 mM dithiothreitol (DTT, 18064014, Invitrogen) in wash buffer). The centrifuge tube then was shocked in an ice bath for 1 h to disintegrate the tissue. After these steps, we placed the tissue pieces in a petri dish and squeezed them to isolate the glands using a glass slide. We resuspended the glands in advanced DMEM/F12 (12630‐010, Gibco) with 10% fetal bovine serum (FBS; SE200‐ES, VISTECH) and collected them into a tube. We centrifuged the tube for 5 min at 300*g* and washed the glands for two times. The glands were digested with TrypLE (12604‐021, Gibco) at 37°C for 5 min for subsequent culture.

### 2.2 Culture medium composition

We used six culture media in this study and observed the morphology of cells using an inverted microscope. The culture medium 1 (Chem.1) was advanced DMEM/F12 medium with 10% FBS, 100 units/ml penicillin and 100 μg/ml streptomycin, 1× GlutaMax (35050‐061, Gibco), and 1× N‐2‐hydroxyethylpiperazine‐N‐2‐ethane sulfonic acid (HEPES; 15630‐080, Gibco). The culture medium 2 (Chem.2) was advanced DMEM/F12 medium with 1× B27 supplement (17504‐044, Gibco), 1× N‐2 supplement (17502‐048, Gibco), 100 units/ml penicillin and 100 μg/ml streptomycin, 1× GlutaMax, and 1× HEPES. The culture medium 3 (Chem.3) was the same as Chem.2, except that 200 ng/ml Noggin (#120‐10C, PeproTech) and 100 ng/mL epidermal growth factor (EGF; #AF‐100‐15, PeproTech) were added.^14^ The culture medium 4 (Chem.4) was Chem.2 with 1 mM N‐acetylcysteine (A9165, Sigma‐Aldrich), 50 ng/ml EGF, 100 ng/ml Noggin, 100 μg/ml primocin, 200 ng/ml fibroblast growth factor 10 (FGF10; #100‐26, PeproTech), 1 nM gastrin (G9145, Sigma‐Aldrich), 2 μM A83‐01 (2939, Tocris Bioscience) and 10 mM nicotinamide (N0636, Sigma‐Aldrich). The culture medium 5 (Chem.5) was the same as Chem.4, except that 500 ng/ml R‐spondin1 (#120‐38, PeproTech) and 100 ng/ml Wnt3A (5036‐WN, R&D Systems) were added. The conditioned medium (Cond.) was advanced DMEM/F12 medium with 50% Wnt3A, R‐spondin1 and Noggin conditioned medium, 1× B27 supplement, 1× N‐2 supplement, 100 units/ml penicillin and 100 μg/ml streptomycin, 1× GlutaMax, 1× HEPES, 1 mM N‐acetylcysteine, 50 ng/mL EGF, 100 μg/ml primocin, 200 ng/ml FGF10, 1 nM gastrin, 2 μM A83‐01 and 10 mM nicotinamide. And the Wnt3A, R‐spondin1 and Noggin conditioned medium was generated from L‐WRN cells (CRL‐3276, ATCC) according to the manufacturer's protocol. The chemically defined medium we used for subsequent culture of gastric epithelial cells is Chem.5.

### 2.3 2D monolayer culture on Matrigel

First, we coated four‐well plates with 250 μl Matrigel (356231, Corning) diluted in DPBS (1:40) per well at 37°C for more than 3 h. Then, we seeded 100,000 cells on Matrigel‐coated plates and for each well, cultured in 500 μl culture medium. The medium was changed on the second day after seeded and every 3 days thereafter. And 10 μM Y‐27632 (S1049, Selleckchem) was added for the first 3 days. Some points that should be noted: for primary gland cells, Cond. with 10 μM Y‐27632 was used for the initial 5 days, followed by Chem.; and for other non‐primary cells, both Chem. and Cond. can be used according to study requirements. The above mentioned culture system was named gel monolayer (GM). Another 2D culture was the same as the above method, except that the plates were not coated by Matrigel.

Passaging was performed when the cell coverage was greater than 80%. The culture medium was discarded and the monolayer cells were rinsed with DPBS. About 200 μl TrypLE was then added to plates per well, and the plates were incubated at 37°C for 10 min to completely digest cells. After these, we used 500 μl advanced DMEM/F12 to terminate the digestion. Cells were spun down at 500*g* for 5 min and resuspended for subculture. The steps of freezing and thawing were consistent with the conventional method. The frozen medium was 10% dimethylsulfoxide (DMSO; D2650, Sigma‐Aldrich) in FBS.

### 2.4 3D organoid culture

For each well of 96 well‐plates, 2500–5000 cells were suspended in culture medium and mixed with five times the volume of Matrigel. The total volume was 8 μl per well. The cell suspension was added to 96‐well plates and incubated at 37°C for 15 min to solidify the Matrigel. The cells were cultured in 200 μl medium per well, and other culture conditions were the same as 2D monolayer culture.

### 2.5 Cell viability assay

We compared the cell viability of 3D, 2D and gel monolayer (GM) cultures. The same number of cells were seeded into 96‐well plates. And cell viability was detected using the CellTiter‐Glo 3D Reagent (G968B, Promega) according to the manufacturer's instructions on Days 0, 3, 6, 9 and 15. The results were analysed by GraphPad Prism 6.

### 2.6 Immunofluorescence

We placed a glass slide into each well of four‐well plates and coated the plates with Matrigel. Then, cells were cultured according to the 2D culture method as above mentioned. When the cell coverage reached 60%, the cell slides could be used for immunofluorescence staining. Cells were fixed in 4% paraformaldehyde (P1110, Macgene) for 30 min, followed by permeabilizing with 0.5% Triton X‐100 (T9284, Sigma‐Aldrich) for 20 min at room temperature. The cells were blocked with 3% bovine serum albumin (130‐091‐376, MiltenyiBiotec) in DPBS for 1 h at 37°C or overnight at 4°C. The cells were incubated in primary antibodies at 4°C overnight, and stained with Hochest 33342 (1:500) (H3570, Invitrogen) and secondary antibodies for 4 h at room temperature. Primary antibodies were rabbit anti‐FABP5 (1:250) (ab255276, Abcam), mouse anti‐E‐cadherin (1:200) (ab76055, Abcam), mouse anti‐NME1 (1:500) (MA5‐15642, Invitrogen), rabbit anti‐E‐cadherin (1:500) (ab40772, Abcam), rabbit anti‐MUC5AC (1:250) (ab3649, Abcam), mouse anti‐MUC6 (1:200) (sc33668, Santa Cruz Biotechnology) and rabbit anti‐MKI67 (1:250) (ab16667, Abcam). The secondary antibodies were donkey anti‐rabbit immunoglobulin G conjugated with Alexa Fluor 488 (1:500) (ab150073, Abcam) and goat anti‐mouse immunoglobulin G conjugated with Alexa Fluor 568 (1:500) (A11004, Thermo Fisher Scientific). Finally, slides were sealed and imaged by fluorescence microscopy.

### 2.7 Effects of medium components on cultured cells

To evaluate the effects of medium components on cells, we analysed changes in cell morphology and gene expression by adding or removing a component based on Chem. We added CHIR99021 (S2924, Selleckchem), valproic acid (VPA, S3944, Selleckchem), or both CHIR99021 and VPA to the culture cells, respectively. The cells were dissociated into single‐cell suspensions and stained with 7‐AAD viability staining solution (420403, BioLegend) for fluorescence‐activated cell sorting (FACS; BD FACS ARIA SORP, BD Biosciences) on Days 6 and 14. Wnt3A, R‐spondin1, Noggin, A83‐01 or EGF were removed from Chem. to form minus culture medium. As above mentioned, the cells were digested for FACS on Days 3, 6 and 10. 7‐AAD^−^ cells were sorted into strip tubes with 8 μl lysis buffer by FACS. The lysis buffer contained 1 U/μl RNase Inhibitor (2313A, Takara), 0.475% Triton X‐100, 2.5 μM oligo dT primer and 2.5 mM dNTP (4019, Takara). There were three replicates per sample for subsequent bulk RNA sequencing library construction.

### 2.8 Single‐cell transcriptome sequencing

Single‐cell transcriptome sequencing libraries were prepared according to the previously published methods.^15^ Human gastric glands and cultured cells were dissociated into single cell suspensions and sorted into 96‐well plates containing 2 μl lysis buffer. SuperScript II reverse transcriptase (18064014, Invitrogen), template switch oligo primer, and barcoded primers with unique molecular identifiers (UMIs) were used to reverse transcribe mRNAs, followed by cDNAs amplification and purification. After being amplified using a biotin‐anchored primer with index tags, cDNAs were fragmented and enriched for library construction. The final libraries were constructed by KAPA Hyper Prep Kits (KK8504, KAPA Biosystems), and sequenced on Illumina HiSeq 4000 platform or Illumina NovaSeq 6000 platform (Novogene or Anoroad).

### 2.9 Bulk RNA sequencing

There were two kinds of methods to prepare bulk RNA libraries. For 100 cells sorted into strip tubes, reverse transcription and cDNAs amplification were the same as single‐cell transcriptome sequencing library preparation, except that the reagent volume was doubled. Then the libraries were constructed by TruePrep DNA Library Prep Kit V2 (TD502‐02, Vazyme) for Illumina. For precipitation of the cultured cells, RNAs were extracted using RNeasy Mini Kit (74506, Qiagen) and mRNAs were isolated from total RNAs by NEBNext Poly(A) mRNA Magnetic Isolation Module (E7490L, New England Biolabs). The transcriptome libraries were prepared using NEBNext Ultra II RNA Library Prep Kit (E7770L, New England Biolabs) for Illumina.

All these kits were applied according to the manufacturer's instructions. The library sequencing was performed on the Illumina HiSeq 4000 platform or Illumina NovaSeq 6000 platform on the paired‐end 150‐bp mode.

### 2.10 Real‐time quantitative PCR

The RNAs of cultured cells were extracted using RNeasy Mini Kit. We used First Strand cDNA Synthesis SuperMix (E042, Novoprotein) to reverse transcribe RNAs and to remove genome DNA. And we used ChamQ Universal SYBR qPCR Master Mix (Q711, Vazyme) to perform qPCR according to the manufacturer's instructions with *GAPDH* as an internal control.

### 2.11 Processing of transcriptomic data

For STRT scRNA‐seq data set, we utilized UMI‐tools to extract barcodes and UMIs from the R2 reads.^16^ The template switch oligo and polyA tail sequence were removed from the obtained reads. Besides, reads with low‐quality bases were also discarded using seqtk (https://github.com/lh3/seqtk). Next, we aligned the obtained clean reads to the human GRCh38 genome by STAR,^17^ and used featureCounts^18^ to calculate the uniquely mapped reads and UMI‐tools to quantify the UMIs. Based on the obtained UMI expression matrix, we filtered cells with <1000 detected genes, <10,000 detected transcripts and high mitochondrial gene‐expression fractions. Harmony was used to reduce the batch effect (https://github.com/immunogenomics/harmony),^19^ and Seurat (https://satijalab.org/seurat/) was used to identify highly variable genes, dimensionality reduction analysis and clustering.^20^

For bulk RNA‐seq data set, the trimming and quality control of the raw fastq files were fulfilled by TrimGalore (https://github.com/FelixKrueger/TrimGalore). Then, the trimmed fastq files were aligned to the human GRCh38 genome using STAR. RSEM was used to quantify the gene/transcript abundances.^21^

For scRNA‐seq data set, we identified DEGs for each cell cluster with the FindAllMarkers function in Seurat. For bulk RNA‐seq data set, we conducted the DEG analysis with DE‐seq2.^22^ We utilized clusterProfiler to perform enrichment analysis.^23^ All the statistical analyses were performed and related figures were generated in R software.

### 2.12 Calculation of the cell cycling score

We downloaded two curated cell cycle gene sets representing G1/S phase and G2/M phase, respectively.^24^ Then, we used these two gene sets to calculate the cycling score with AUCell,^25^ and generated a G1/S score and a G2/M score for each sample.

## 3 RESULTS

### 3.1 The establishment of a new 2D culture system for hGEPL cell lines

**FIGURE 1:**
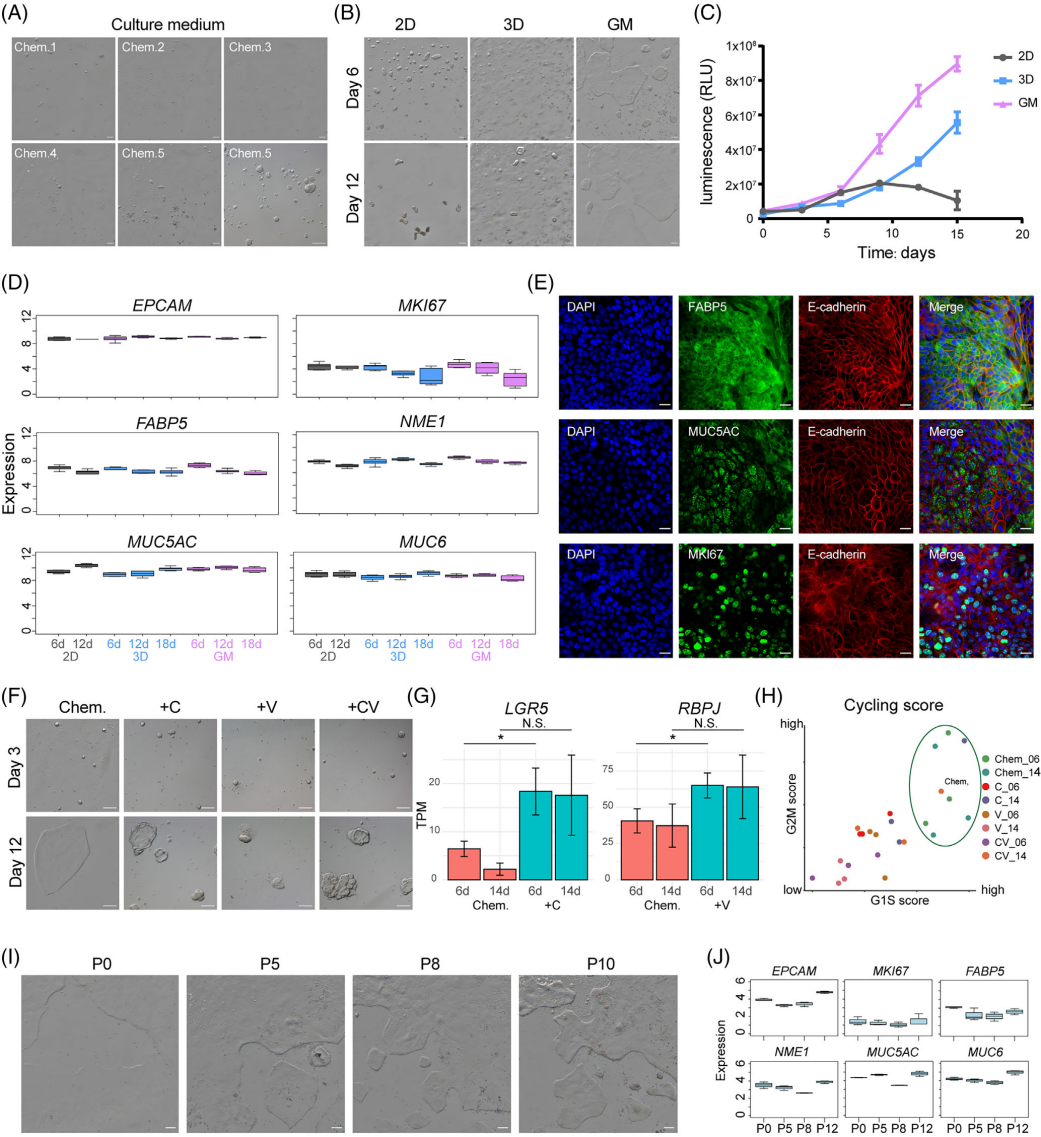
Culture system establishment of human gastric epithelial progenitor‐like (hGEPL) cells. (A) Primary human epithelial cells cultured in different media on Day 6. Scale bar: 100 μm. (B) Primary human epithelial cells cultured in 2D, 3D and GM modes. Scale bar: 100 μm. (C) Cell proliferation analysis for primary human epithelial cells cultured in 2D, 3D and GM modes after serial culture. (D) The expression levels of representative genes in primary human epithelial cells cultured in 2D, 3D and GM modes after serial culture. (E) Immunofluorescence staining of *FABP5*, *MUC5AC* and *MKI67* on hGEPL cell line. Scale bar: 20 μm. (F) hGEPL cell line cultured in different culture media (Chem, Chem+C, Chem+V and Chem+CV). Scale bar: 100 μm. (G) The expression levels of *LGR5* and *RBPJ* in hGEPL cell line cultured with Chem+C and Chem+V, respectively. Significance was determined by *t*‐tests. *p value < 0.05. (H) Cycling score of hGEPL cell line cultured in different culture media (Chem, Chem+C, Chem+V and Chem+CV). (I) hGEPL cells after serial passages. Scale bar: 100 μm. (J) The expression levels of representative genes in hGEPL cells after serial passages.

To expand human adult gastric epithelial cells on a large scale in vitro, we first tested five commonly used gastric epithelial culture media (Chem.1–Chem.5, see Section 2 for details). The cellular states and proliferation rates of human adult gastric epithelial cells varied dramatically in different media (Figure 1A). Among them, Chem.1, Chem.2 and Chem.3 were not able to maintain the normal growth of primary human adult gastric epithelial cells, while epithelial cells in Chem.4 and Chem.5 could form mono‐colony, and more colonies were formed in Chem.5. After 6 days of culture, colonies of up to ~100 μm diameter could be formed in Chem.5 (Figure 1A). These results indicated that Chem.5 medium had stronger colony formation and proliferation ability, which was more suitable for the culture of primary human adult gastric epithelial cells than the other four media.

Based on the Chem.5 medium (referred to as Chem. in the following description), we next explored the effects of different culture modes on the primary human gastric epithelial cells. We tested conventional 2D and 3D organoid culture modes, and found that cells in conventional 2D culture mode would form round colonies, but could be only maintained for about 15 days in culture and then gradually die (Figure 1B,C). Under traditional 2D culture mode, the support matrix is missing, which is very different from the physiological environment in vivo, and cells lose the ability to proliferate after several divisions and then undergo cellular senescence.^26^, ^27^ Our results are also consistent with the previous study that primary human gastric epithelial cells can only be cultured for 2 weeks and cannot be further passaged.^28^ For 3D organoid culture mode, although cell proliferation was able to be maintained for a long time in culture, the cells proliferated quite slowly (Figure 1C). To avoid the drawbacks of conventional 2D and 3D organoid culture modes, we combined them by first coating petri dishes with thin Matrigel, and then cultured the cells on the surface of the gel, termed as GM culture mode. This GM culture mode achieved great culture results: the gastric epithelial cells from human adults first grew as round colonies, then stretched, and proliferated at a much faster rate than in conventional 2D or 3D organoid culture modes (Figure 1C). Therefore, GM culture mode was easy to operate and the human adult epithelial cells proliferated much faster, which integrated the advantages of the other two culture modes and achieved optimal growth in our hands.

Next, we conducted transcriptomic analysis to make comparisons among 2D, 3D and GM culture modes. As shown in Figure 1D, epithelial (*EPCAM*), cell cycle (*MKI67*), stemness‐related (*FABP5*, *NME1*) and gastric epithelial cell differentiation genes (*MUC5AC*, *MUC6*) were expressed at similar levels among these different culture modes. We also found that the results of qPCR were similar to those of the transcriptomics results, and the cells in GM culture mode were less differentiated (Figure S1A). Meanwhile, immunofluorescence staining also indicated that cells in GM culture mode actively proliferated and expressed well‐known marker genes of gastric epithelial cells (Figure 1E, Figure S1B). We also compared the differentially expressed genes (DEGs) under these three culture modes and conducted a Gene Ontology analysis (GO). We found that the DEGs of GM culture mode were mainly enriched in cell proliferation, amino acid transport and metabolism (Figure S1C,D).

Previous studies reported two molecules, CHIR99021 (CHIR or C, activator of the WNT pathway) and valproic acid (VPA or V, activator of the NOTCH pathway), which could enhance the self‐renewal ability of mouse intestinal stem cells (ISCs), and the colony formation ability was 100 times higher when these two molecules were applied.^29^ Tong et al. also found that the addition of CHIR and VPA (CV) supported the growth of mouse and human intestinal epithelial cells on 2D collagen‐based monolayer culture system, and promoted the enrichment of *Lgr5* ^+^ population.^11^ Therefore, we separately added C, V or CV to Chem. medium to expand the primary human gastric epithelial cells using the GM culture mode. The addition of either CHIR or VPA increased the expression levels of WNT signalling pathway‐related gene *LGR5* and NOTCH signalling pathway‐related gene *RBPJ*, respectively (Figure 1G). However, these two molecules did not enhance the ability of colony formation in the GM culture system. Even worse, cells cultured for 10 days with these two molecules added slowed down their proliferation, and the cell morphology also became abnormally thickened (Figure 1F,H). Thus, the effect of CHIR and VPA for the primary human adult gastric epithelial cells was quite different compared with their beneficial effects for the self‐renewal of ISCs.

Next, we assessed the transcriptomes for different passages of the cells in GM culture system. Previous work has shown that long‐term culture of primary intestinal epithelium as monolayer is very difficult due to the rapid loss of stem cells and their accelerated apoptosis, and an important limitation of all monolayer culture systems that need to be solved is the difficulty to passage and propagate the cells,^30^ and long‐term passage and culture needs to be further optimized.^10^, ^12^, ^31^ Through our optimized culture system, the gastric progenitor cells can be continuously passaged for 12 passages and can last for 5 months. As the passage number increased, the cultured cells remained stable for the specific expression signatures of gastric epithelial cells, and the morphologies of the colonies were very similar to those of the early primary culture (Figure 1I,J). In summary, we established a monolayer culture system that used GM culture modes with Chem. medium to expand primary human adult gastric epithelial cells in vitro.

### 3.2 Comparison between chemically defined medium and conditioned medium for hGEPL cell lines

**FIGURE 2:**
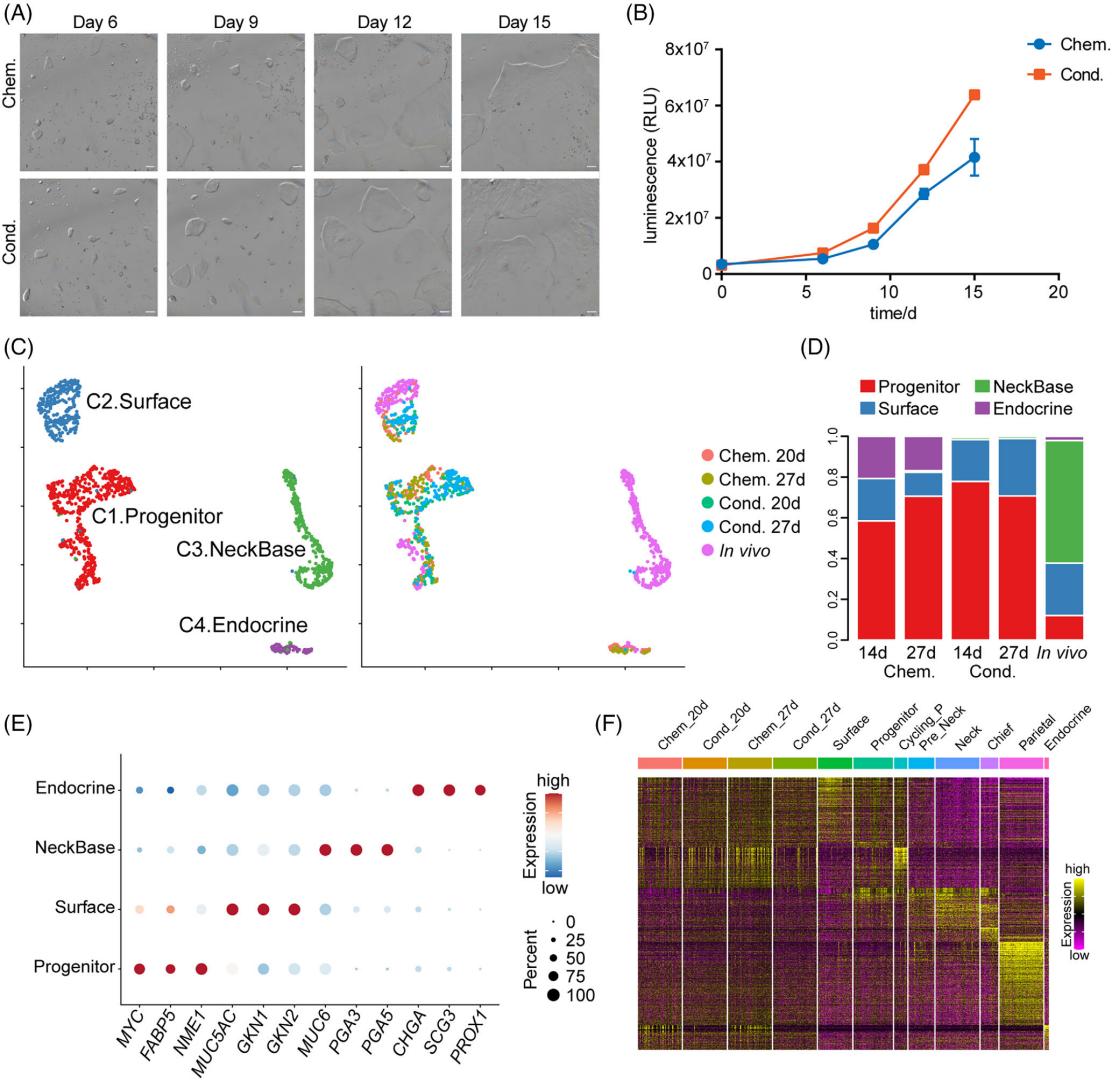
Comparison between hGEPL cells cultured in Chem. and Cond. media. (A) hGEPL cell line cultured in Chem. and Cond. media after serial culture. Scale bar: 100 μm. (B) Cell proliferation analysis for hGEPL cell line cultured in Chem. and Cond. media after serial culture. (C) UMAP plots exhibiting clustering (left) and sample information (right) of in vivo human gastric epithelial cells and hGEPL cells cultured in Chem. and Cond. media. (D) Cluster ratios of hGEPL cells cultured in Chem. and Cond. media. (E) Dot plots exhibiting the expression levels of representative genes in each cluster. The colour key from blue to red indicates low to high expression levels, respectively. The circle size indicates the percentage of cells expressing a certain gene. (F) Heatmap exhibiting gene expression patterns in cultured hGEPL cells and in vivo gastric corpus cells using the DEGs of in vivo gastric corpus cell types. The colour key from purple to yellow indicates low to high expression levels, respectively. DEG, differentially expressed gene; hGEPL, human gastric epithelial progenitor‐like.

Although the Chem. medium combined with GM culture system can expand primary human adult gastric epithelial cells efficiently, it is a chemically defined medium, which relies on very expensive purified growth factors. Thus, we tested a conditioned medium (Cond.)^32^ based on a supportive cell line that secreted three growth factors (WNT, R‐spondin1 and Noggin), and made comparisons between the chemically defined medium (Chem.) and conditioned medium (Cond.). We found that the morphologies of the colonies were similar in these two media. Colonies formed about 6 days after being passaged, and the colonies could grow to full on the petri dish after around 15 days after passage (Figure 2A). In addition, the gastric progenitor cells cultured in Cond. medium had a faster growth rate than those in Chem. medium (Figure 2B).

To analyse the cellular composition of the human gastric epithelial cells cultured in Chem. and Cond., we profiled the transcriptomes using single‐cell RNA sequencing (scRNA‐seq) technique and used in vivo normal gastric epithelial cells as reference. After removing batch effects, we found that cells in Chem. and Cond. media were mixed together very well, and were clustered together with the in vivo gastric progenitor and mucous surface cells (Figure 2C). The expression patterns of representative marker genes also supported the result (Figure 2E). For different days during the culture, the expression patterns of gastric epithelial cell marker genes were stable for both Chem. and Cond. culture conditions (Figure S2A–C). Compared with Cond. medium, there were some endocrine cells in the Chem. medium (Figure 2C,D). Interestingly, the proportion of progenitor cells was much higher in both culture conditions compared with the in vivo situation (Figure 2D). Consistently, the expression patterns of the cultured cells were also more similar to those of *in vivo* gastric epithelial progenitor cells, thus we termed the cultured cells as human gastric epithelial progenitor‐like (hGEPL) cells (Figure 2F).

To sum up, the gene expression signatures of the hGEPL cells were similar between Chem. and Cond. culture media, while the proliferation rate was higher in Cond. medium. Thus, if large‐scale expansion of the hGEPL cells is needed, we recommend Cond. medium, which is cheaper and the cells grow faster to obtain sufficient amounts of materials for large‐scale experiments within a shorter time. However, in the subsequent context, since we wanted to explore the effects of key niche factors in the Chem. medium on hGEPL cell line, we adopted the following strategy: first, cultured the hGEPL cells in Cond. medium for 5 days for the initial colonies, and then changed the medium to Chem. medium to identify the function of each individual niche factor.

### 3.3 BMP and WNT signalling pathways play important roles in the differentiation of hGEPL cells

**FIGURE 3:**
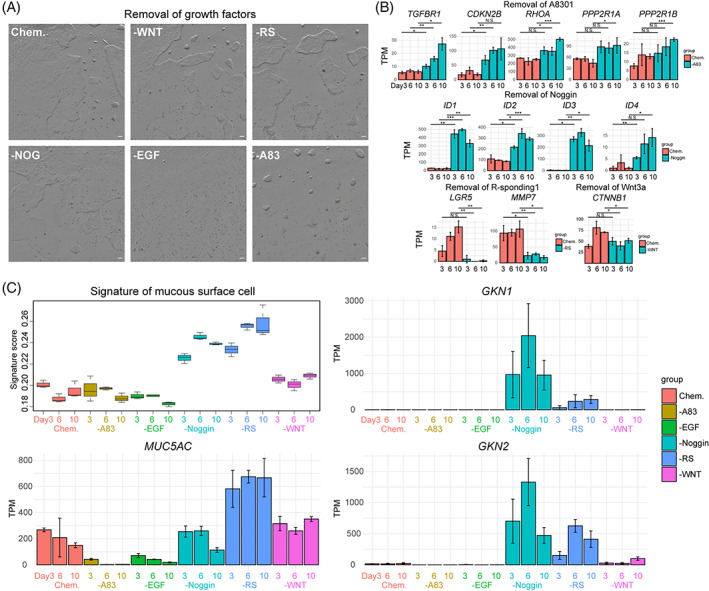
Effects of growth factors on hGEPL cells. (A) hGEPL cell line cultured in Chem. and after the removal of a certain growth factor. Scale bar: 100 μm. WNT, WNT3a; RS, R‐spondin1; NOG, Noggin; A83, A83‐01. (B) The expression levels of representative genes in hGEPL cells after the removal of a certain growth factor. (C) The signature score and expression levels of representative genes of mucous surface cells in hGEPL cells after the removal of a certain growth factor. A83, A83‐01; RS, R‐spondin1; hGEPL, human gastric epithelial progenitor‐like.

For the Chem. culture medium, several niche factors are indispensable for the self‐renewal and proliferative capacity of hGEPL cells, such as EGF, WNT3a, R‐spondin1, Noggin and A83‐01 (inhibitor of TGF‐β signalling pathway).^7^, ^33^, ^34^, ^35^ To explore their roles in maintaining the homeostasis of hGEPL cells, we individually removed them from the Chem. culture medium. As shown in Figure 3A, the removal of different niche factors resulted in different growth states of hGEPL cells. After the removal of WNT3a or R‐spondin1, the growth rate of hGEPL cells decreased drastically, and the colonies became slightly thicker. After the removal of Noggin, the colonies became stretched. EGF was essential for the growth of hGEPL cells, and most hGEPL cells died after removing it. After the removal of A83‐01, the morphologies of the colonies became shrunken and thickened, and the number of the colonies also decreased (Figure 3A).

At the molecular level, after the removal of A83‐01, the inhibition of TGF‐β signalling pathway was relieved, the expression level of TGF‐β receptor gene (*TGFBR1*) was upregulated, and the downstream target genes of TGF‐β pathway were also upregulated as expected (*CDKN2B*, *RHOA*, *PP2R1A* and *PP2R1B*) (Figure 3B). The removal of Noggin led to the activation of BMP signalling pathway, and the expression levels of BMP signalling target genes *ID1*, *ID2*, *ID3* and *ID4* were upregulated (Figure 3B). After the removal of R‐spondin1 or WNT3a, the WNT signalling pathway was suppressed, and the expression levels of its target genes *LGR5*, *MMP7* and *CTNNB1* were downregulated (Figure 3B).

To evaluate the effects of these niche factors and small molecules on hGEPL cells' self‐renewal and differentiation, we profiled the transcriptomes of hGEPL cells after the removal of the factors, and then calculated their signature scores compared to in vivo human gastric epithelial cell types. Interestingly, we found that the signature scores of mucous surface cells dramatically increased after Noggin or R‐spondin1 were removed, and the corresponding marker genes of mucous surface cells such a*s GKN1*, *GKN2* and *MUC5AC* were significantly upregulated (Figure 3C). After removing WNT3a, the hGEPL cells also showed a tendency to differentiate into mucous surface cells (Figure 3C). We also explored whether there was the differentiation of other mature gastric epithelial cell types after niche factor removal, but the relevant characteristics were not significant, indicating that the differentiation of hGEPL cells to mature cell types other than mucous surface cells may be controlled by other additional factors (Figure S3).

These results indicated that these niche factors play important roles in the homeostasis of hGEPL cells, and the removal of them may result in premature differentiation. The block of BMP signalling pathway and activation of WNT signalling pathway play decisive roles in maintaining the self‐renewal ability of the hGEPL cells and preventing them to prematurely differentiate into mucous surface cells.

### 3.4 Identification of key regulatory transcription factors in the differentiation of hGEPL cells

**FIGURE 4:**
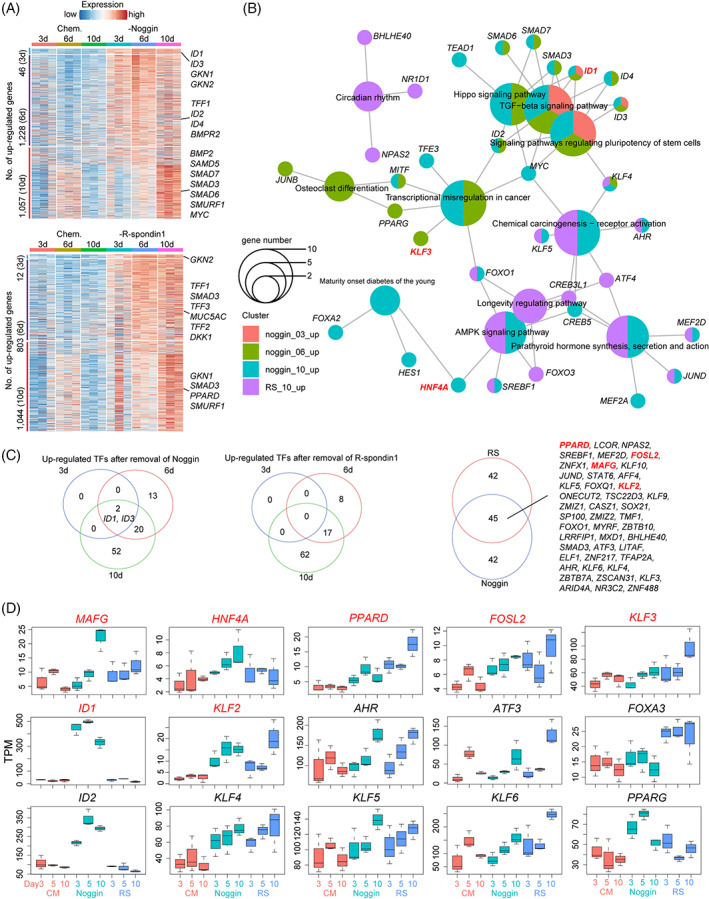
Roles of Noggin and R‐spondin1 in hGEPL cells. (A) Heatmap exhibiting the upregulated DEGs after 3, 6 and 10 days of Noggin (up) or R‐spondin1 (bottom) removal in hGEPL cell line. The colour key from blue to red indicates low to high expression levels, respectively. (B) Enriched KEGG pathways using the upregulated DEGs after 3, 6 and 10 days of Noggin (up) or R‐spondin1 (bottom) removal in hGEPL cell line. (C) Venn diagrams exhibiting upregulated TFs after the removal of Noggin or R‐spondin1. (D) The expression levels of representative TFs in hGEPL cells after the removal of Noggin or R‐spondin1. RS, R‐spondin1; DEG, differentially expressed gene; hGEPL, human gastric epithelial progenitor‐like; TF, transcription factor.

Next, we explored the dynamic expression patterns after withdrawing Noggin or R‐spondin1. For both Noggin and R‐spondin1, in the first 3 days of withdrawal, the differentiation was only slightly increased, and the number of DEGs was also small compared with the control (cells cultured in Chem. medium). However, from the 6th day to 10th day after Noggin or R‐spondin1 removal, the differentiation strongly accelerated, and the number of DEGs also increased sharply (Figure 4A). After noggin removal, representative marker genes of mucous surface cells (*GKN1*, *GKN2* and *TFF1*), the receptor genes of BMP pathway (*BMPR2*), and the target genes of BMP signalling pathway (*ID1/2/3/4*, *SMAD3/5/6/7* and *SMURF1*) were upregulated. Similarly, after removal of R‐spondin1, markers of surface mucous cells (*GKN1/2*, *TFF1/2* and *MUC5AC*) and transcription factors of the WNT signalling pathway (*PPARD*) were upregulated. Interestingly, BMP‐related genes (*SMAD3* and *SMURF1*) were also upregulated (Figure 4A). Since transcription factors (TFs) play crucial roles for cell differentiation, we then focused on the dynamic changes of TFs during the differentiation of hGEPL cells into mucous surface cells. After the removal of Noggin, the expression levels of *ID1*, *ID3* and *ID4* firstly increased, indicating that the BMP signalling pathway was activated, and the hGEPL cells start differentiation, and TFs such as *MYC* and *PPARG* were also upregulated (Figure 4B). Although no TF‐related terms were enriched in the first 6 days after the removal of R‐spondin1, the TF enriched by R‐spondin1 and Noggin removal appeared to coincide greatly at Day 10 (Figure 4B,C). This result implied that after the removal of either R‐spondin1 or Noggin, the hGEPL cells start differentiation, and they differentiate into comparable types of mature cells at the later stage.

After removing Noggin, the cellular state gradually changed, *ID1* and *ID3* were continuously upregulated (Figure 4B). After the removal of R‐spondin1, the change of the cellular state is initially slow but accelerated later (Figure 4A,C). During the differentiation of hGEPL cells into mucous surface cells, the removal of Noggin and R‐spondin1 shared 45 upregulated transcription factors, suggesting that these TFs may be more universal for the mucous surface cell differentiation (Figure 4C). We also compared these identified TFs with the TFs important for mucous surface cell differentiation in vivo, and found that several TFs were shared between them, such as *MAFG*, *HNF4A*, *PPARD*, *FOSL2*, *KLF3*, *ID1* and *KLF2* (Figure 4C,D).

## 4 DISCUSSION

Although the identity of stem cells in the small intestine has been thoroughly studied,^36^, ^37^ the identity and location of gastric stem/progenitor cells are still controversial.^38^, ^39^, ^40^ Considering the difficulties of studying human gastric epithelial stem/progenitor cells in situ, many efforts have been made to understand them by in vitro culture system. Bartfeld et al. first cultured human adult gastric organoids, where gastric epithelial stem/progenitor cells can be differentiated into surface mucous cells, neck mucous cells, chief cells, and a small number of endocrine cells.^7^ McCracken et al. first induced embryonic stem cells (ESCs) into gastric antrum epithelial structure.^41^ These two studies laid the foundation for different culture methods of human gastric organoids. However, the research on physiological functions of human adult gastric organoids is limited and the operation is difficult, and other primary cell culture systems are also being developed.

In recent years, 2D cell culture has flourished. Liu et al.^10^ developed a 2D culture method for *Lgr5+* stem cells from the small intestine. They were surprised to find that the proportion of ISCs in 2D culture was significantly higher than that in 3D organoid culture, and the intestine epithelial cells cultured in monolayer could effectively form 3D organoid after being suspended in Matrigel. Boccellato et al.^42^ regenerated the columnar epithelium of human gastric mucosa using the air–liquid interface (ALI) culture method. Compared with the standard ALI culture, the gastric polarized epithelial monolayer consists of highly polarized cells of all gastric gland lineages. The cellular states and interactions can be better observed in the 2D monolayer culture system. Furthermore, in contrast to the inaccessibility of the apical epithelium region in organoids, monolayer culture system is more favourable for imaging, genetic manipulation and provides a suitable system for studying pathogen–epithelial interactions. We established a 2D monolayer culture system using Chem. medium and GM culture mode to maximize cell proliferation and maintain characteristic stability. The cells in our cultured system clustered together with in vivo gastric stem/progenitor cells and had similar gene expression patterns (Figure 2C,F). The proportion of stem/progenitor cells in the total cell population increased drastically compared with in vivo situations (Figure 2D). All of these results indicate that we have successfully established hGEPL cell lines.

Although niche factors of WNT, BMP, NOTCH and EGF signalling pathways are indispensable for the self‐renewal and proliferative capacity of gastrointestinal epithelial cells, their exact roles remain elusive. Our study showed that activation of WNT signalling pathway and repression of BMP signalling pathway were critical for maintaining the self‐renewal ability of hGEPL cells, and cells differentiated into gastric mucous surface cells after blocking WNT or activating BMP signalling pathway (Figure 3C), consistent with previous reports in 3D culture.^7^ Interestingly, we found that removing only a single factor in the WNT pathway, WNT3a or R‐spondin1, could induce differentiation of hGEPL cells, and R‐spondin1 played a more dominant role than WNT3a (Figure 3C). For the BMP signalling pathway, after Noggin removal, the expression of BMP target gene *ID1* increased as expected (Figure 3B, Figure 4A,B), indicating the activation of BMP signalling pathway and the shift of cell identity to mucous surface cells, which was consistent with the recently published study.^35^ For the NOTCH pathway, after its activation with VPA, the proliferation of hGEPL cells became slow (Figure 1G,H), which was contrary to the intestinal epithelial cells, reflecting the physiological differences between stomach and intestine.^29^ For EGF pathway, we found that once EGF was removed (Figure 3A), most hGEPL cells could not survive, which also indicated the importance of EGF for the self‐renewal of hGEPL cells.

It is very difficult to expand the culture of adult primary cells and enrich for stem/progenitor cell types in vitro. How to better maintain the self‐renewal and differentiation potential of hGEPL cells is the focus of our future research direction. The stem cell niche is the local tissue microenvironment that regulates the self‐renewal and differentiation of stem cells, and is crucial for the maintenance of normal physiological functions of stem cells.^43^ The function of stem cells is controlled by multiple niche signalling pathways. How to better simulate the real niche signalling pathways in vitro has always been a great challenge. Sato et al.^36^ induced organoids into undifferentiated state to maintain their potential of amplification. Barker and Huch et al.^6^, ^44^ performed differentiation‐promoting treatment to generate nascent differentiated cells. Fujii et al.^45^ established a new culture condition for intestinal epithelial cells, enabling human intestinal organoids to undergo simultaneous multi‐lineage differentiation and self‐renewal. Yin et al.^29^ screened out two small molecules, CHIR99021 and valproic acid, to synergistically maintain the self‐renewal of mouse intestinal stem cells, resulting in homogeneous cultures. Exosomes are also critical to the regulation of stem cell niches. Exosomes are transport vesicles secreted by cells, which are important mediators for information transfer and material exchange between cells.^46^ Exosomes secreted by stem cells are often involved in regulating the microenvironment and immune system between cells, mediating extensive communications between cells, and participating in the physiological processes of cells and tissues.^47^, ^48^ The regenerative potential of exosomes derived from MSCs has been demonstrated in various model diseases such as kidney, liver, heart and nerve injury.^49^ miRNAs in exosomes of neural progenitor cells are closely related to cell growth and apoptosis.^50^ ESC‐derived exosomes can activate the proliferation of cardiomyocytes in damaged cardiac tissue to reduce the inflammatory response.^51^ Understanding the biological characteristics of exosomes derived from stem cells will provide a new perspective for better understanding and regulating the physiological functions of stem cells.

In addition to the niche and exosomes of stem cells, the selection of novel biomaterials is also an important way to enhance the bioactivity and function of hGEPL cell lines, thus more accurately representing the characteristics of gastric tissue. The culture system we developed currently uses Matrigel to maintain the normal growth of hGEPL cells. But Matrigel is derived from mouse sarcoma cells, which exhibits batch‐to‐batch variability and it is difficult to control its physical and chemical properties. And Matrigel's murine origin hinders the use of organoids in human clinical transplantation.^52^, ^53^ Novel synthetic hydrogels have become potentially a better choice, which can mimic salient elements of the natural extracellular matrix, and can support cell adhesion.^54^ Synthetic hydrogel supports the growth and differentiation of human and mouse intestinal stem cells into organoids.^55^ Studies have shown that,^56^ when Phosphorene coating is applied to hydrogel, the osteogenic ability of human dental pulp stem cells is enhanced. There are also studies combining borophene with hydrogel‐based substrates, which is considered to be a highly sensitive method that can be used as a biodetector for clinical translational medicine.^57^

There are three key characteristics of our hGEPL cells. First, we overcome the large gap between the 2D monolayer culture system and in vivo gastric epithelial cells. hGEPL cells have similar gene expression patterns with the in vivo gastric stem/progenitor cells, recapitulating key properties of in vivo gastric epithelium. Second, in contrast to 3D organoids, our GM culture system can be easily introduced into lumen contents without labour‐intensive microinjection and are suitable for genetic manipulation and high‐throughput imaging analysis. Finally, hGEPL cells have similar gene expression patterns of gastric characteristic genes with those of 3D organoids, and can be easily expanded by suspension and passage like classical cell lines, which is a great improvement over previous works.^10^, ^12^, ^31^, ^58^

We have established a robust monolayer cell culture system for adult gastric epithelium. The hGEPL cell lines have biological features comparable to gastric epithelial stem/progenitor cells in vivo and could proliferate steadily over a long period of time. Activation of the WNT signalling pathway and repression of the BMP signalling pathway are critical for the maintenance of the self‐renewal ability of hGEPL cells.

Our novel culture system lays a solid foundation for the study of the physiological function and molecular mechanism of gastric epithelium, and provides a new selection model for the in vitro drug screening for gastric diseases. Meanwhile, our system has certain limitations. Our culture system is relatively expensive, which limits the application of large‐scale experiments. Besides, cells cultured in our system are more homogeneous than 3D organoid system, so it is difficult to study the interactions between different epithelial cell types. However, our culture system can conduct primary culture of gastric epithelial cells without immortalization and other genetic manipulations, which are necessary for traditional normal 2D cell lines, and can better reflect the real characteristics of human adult gastric epithelial cells in vivo.

## AUTHOR CONTRIBUTIONS

Fuchou Tang and Wei Fu conceived the project. Yuan Gao, Ji Dong, Shuyue Qi and Xin Zhou conducted all the study. Yuan Gao and Shuyue Qi performed the experiments with the help of Xinglong Wu and Wendong Wang. Ji Dong conducted the bioinformatics analyses. Xin Zhou contributed to recruitment of study participants. Fuchou Tang and Yuan Gao wrote the manuscript with help from all of the authors.

## CONFLICT OF INTEREST

The authors declare no conflicts of interest.

## Supporting information


**FIGURE S1** Characterization of hGEPL cells

**FIGURE S2** Differences between hGEPL cells cultured in Chem. and Cond. media

**FIGURE S3** The signature scores of in vivo gastric epithelial cell types in hGEPL cells after the removal of a certain growth factor

## Data Availability

All sequencing data of this study have been deposited in the Genome Sequence Archive at the National Genomics Data Center, Beijing Institute of Genomics, Chinese Academy of Sciences / China National Center for Bioinformation (GSA: HRA002916), and are publicly accessible at https://ngdc.cncb.ac.cn/gsa-human/.

## References

[1] Mahadevan V . Anatomy of the stomach. Surgery. 2014;32(11):571‐574.

[2] Seeley RR , Stephens TD , Tate P . Anatomy and Physiology. Cliffs Notes; 2007.

[3] Lee ER . Dynamic histology of the antral epithelium in the mouse stomach: III. Ultrastructure and renewal of pit cells. Am J Anat. 1985;172(3):225‐240. 3993598 10.1002/aja.1001720305

[4] Bhattacharyya A , Chattopadhyay R , Mitra S , Crowe SE . Oxidative stress: an essential factor in the pathogenesis of gastrointestinal mucosal diseases. Physiol Rev. 2014;94(2):329‐354. 24692350 10.1152/physrev.00040.2012 PMC4044300

[5] Gardin C , Bosco G , Ferroni L , et al. Hyperbaric oxygen therapy improves the osteogenic and Vasculogenic properties of mesenchymal stem cells in the presence of inflammation In vitro. Int J Mol Sci. 2020;21(4):1452. 10.3390/ijms21041452 PMC7073059 32093391

[6] Barker N , Huch M , Kujala P , et al. Lgr5(+ve) stem cells drive self‐renewal in the stomach and build long‐lived gastric units in vitro. Cell Stem Cell. 2010;6(1):25‐36. 20085740 10.1016/j.stem.2009.11.013

[7] Bartfeld S , Bayram T , van de Wetering M , et al. In vitro expansion of human gastric epithelial stem cells and their responses to bacterial infection. Gastroenterology. 2015;148(1):126‐136.e126. 25307862 10.1053/j.gastro.2014.09.042 PMC4274199

[8] Schumacher MA , Aihara E , Feng R , et al. The use of murine‐derived fundic organoids in studies of gastric physiology. J Physiol. 2015;593(8):1809‐1827. 25605613 10.1113/jphysiol.2014.283028 PMC4405744

[9] Scott A , Rouch JD , Jabaji Z , et al. Long‐term renewable human intestinal epithelial stem cells as monolayers: a potential for clinical use. J Pediatr Surg. 2016;51(6):995‐1000. 26995514 10.1016/j.jpedsurg.2016.02.074 PMC4921284

[10] Liu Y , Qi Z , Li X , Du Y , Chen YG . Monolayer culture of intestinal epithelium sustains Lgr5(+) intestinal stem cells. Cell Discov. 2018;4:32. 29928510 10.1038/s41421-018-0036-z PMC5997714

[11] Tong Z , Martyn K , Yang A , et al. Towards a defined ECM and small molecule based monolayer culture system for the expansion of mouse and human intestinal stem cells. Biomaterials. 2018;154:60‐73. 29120819 10.1016/j.biomaterials.2017.10.038 PMC5735007

[12] Wang Y , DiSalvo M , Gunasekara DB , et al. Self‐renewing monolayer of primary colonic or rectal epithelial cells. Cell Mol Gastroenterol Hepatol. 2017;4(1):165‐182.e167. 29204504 10.1016/j.jcmgh.2017.02.011 PMC5710741

[13] Wang X , Yamamoto Y , Wilson LH , et al. Cloning and variation of ground state intestinal stem cells. Nature. 2015;522(7555):173‐178. 26040716 10.1038/nature14484 PMC4853906

[14] Broda TR , McCracken KW , Wells JM . Generation of human antral and fundic gastric organoids from pluripotent stem cells. Nat Protoc. 2019;14(1):28‐50. 30470820 10.1038/s41596-018-0080-z PMC7951181

[15] Li L , Dong J , Yan L , et al. Single‐cell RNA‐Seq analysis maps development of human germline cells and gonadal niche interactions. Cell Stem Cell. 2017;20(6):891‐892. 28575695 10.1016/j.stem.2017.05.009

[16] Smith T , Heger A , Sudbery I . UMI‐tools: modeling sequencing errors in unique molecular identifiers to improve quantification accuracy. Genome Res. 2017;27(3):491‐499. 28100584 10.1101/gr.209601.116 PMC5340976

[17] Alexander D , Davis CA , Felix S , et al. STAR: ultrafast universal RNA‐seq aligner. Bioinformatics. 2013;29(1):15‐21. 23104886 10.1093/bioinformatics/bts635 PMC3530905

[18] Yang L , Smyth GK , Wei S . featureCounts: an efficient general purpose program for assigning sequence reads to genomic features. Bioinformatics. 2014;30(7):923‐930. 24227677 10.1093/bioinformatics/btt656

[19] Korsunsky I , Millard N , Fan J , et al. Fast, sensitive and accurate integration of single‐cell data with harmony. Nat Methods. 2019;16(12):1289‐1296. 31740819 10.1038/s41592-019-0619-0 PMC6884693

[20] Satija R , Farrell JA , Gennert D , Schier AF , Regev A . Spatial reconstruction of single‐cell gene expression data. Nat Biotechnol. 2015;33(5):495‐502. 25867923 10.1038/nbt.3192 PMC4430369

[21] Li B , Dewey CN . RSEM: accurate transcript quantification from RNA‐Seq data with or without a reference genome. BMC Bioinform. 2011;12:323. 10.1186/1471-2105-12-323 PMC3163565 21816040

[22] Love MI , Huber W , Anders S . Moderated estimation of fold change and dispersion for RNA‐seq data with DESeq2. Genome Biol. 2014;15(12):550. 25516281 10.1186/s13059-014-0550-8 PMC4302049

[23] Wu T , Hu E , Xu S , et al. clusterProfiler 4.0: a universal enrichment tool for interpreting omics data. Innovation. 2021;2(3):100141. 34557778 10.1016/j.xinn.2021.100141 PMC8454663

[24] Tirosh I , Izar B , Prakadan SM , et al. Dissecting the multicellular ecosystem of metastatic melanoma by single‐cell RNA‐seq. Science. 2016;352(6282):189‐196. 27124452 10.1126/science.aad0501 PMC4944528

[25] Aibar S , Gonzalez‐Blas CB , Moerman T , et al. SCENIC: single‐cell regulatory network inference and clustering. Nat Methods. 2017;14(11):1083‐1086. 28991892 10.1038/nmeth.4463 PMC5937676

[26] Campisi J , di Fagagna FD . Cellular senescence: when bad things happen to good cells. Nat Rev Mol Cell Biol. 2007;8(9):729‐740. 17667954 10.1038/nrm2233

[27] Hayflick L , Moorhead PS . Serial cultivation of human diploid cell strains. Exp Cell Res. 1961;25(3):585‐621. 13905658 10.1016/0014-4827(61)90192-6

[28] Terano A , Mach T , Stachura J , Sekhon S , Tarnawski A , Ivey KJ . A monolayer‐culture of human gastric epithelial‐cells. Digest Dis Sci. 1983;28(7):595‐603. 6861589 10.1007/BF01299919

[29] Yin X , Farin HF , van Es JH , Clevers H , Langer R , Karp JM . Niche‐independent high‐purity cultures of Lgr5+ intestinal stem cells and their progeny. Nat Methods. 2014;11(1):106‐112. 24292484 10.1038/nmeth.2737 PMC3951815

[30] Braverman J , Yilmaz OH . From 3D organoids back to 2D Enteroids. Dev Cell. 2018;44(5):533‐534. 29533766 10.1016/j.devcel.2018.02.016

[31] Beumer J , Artegiani B , Post Y , et al. Enteroendocrine cells switch hormone expression along the crypt‐to‐villus BMP signalling gradient. Nat Cell Biol. 2018;20(8):909‐916. 30038251 10.1038/s41556-018-0143-y PMC6276989

[32] Miyoshi H , Stappenbeck TS . In vitro expansion and genetic modification of gastrointestinal stem cells in spheroid culture. Nat Protoc. 2013;8(12):2471‐2482. 24232249 10.1038/nprot.2013.153 PMC3969856

[33] McCracken KW , Aihara E , Martin B , et al. Wnt/beta‐catenin promotes gastric fundus specification in mice and humans. Nature. 2017;541(7636):182‐187. 28052057 10.1038/nature21021 PMC5526592

[34] Sigal M , Logan CY , Kapalczynska M , et al. Stromal R‐spondin orchestrates gastric epithelial stem cells and gland homeostasis. Nature. 2017;548(7668):451‐455. 28813421 10.1038/nature23642

[35] Wolffling S , Daddi AA , Imai‐Matsushima A , et al. EGF and BMPs govern differentiation and patterning in human gastric glands. Gastroenterology. 2021;161(2):623‐636.e616. 33957136 10.1053/j.gastro.2021.04.062

[36] Sato T , Vries RG , Snippert HJ , et al. Single Lgr5 stem cells build crypt‐villus structures in vitro without a mesenchymal niche. Nature. 2009;459(7244):262‐265. 19329995 10.1038/nature07935

[37] Medema JP , Vermeulen L . Microenvironmental regulation of stem cells in intestinal homeostasis and cancer. Nature. 2011;474(7351):318‐326. 21677748 10.1038/nature10212

[38] Karam SM . Lineage commitment and maturation of epithelial cells in the gut. Front Biosci. 1999;4:D286‐D298. 10077541 10.2741/karam

[39] Hoffmann W . Regeneration of the gastric mucosa and its glands from stem cells. Curr Med Chem. 2008;15(29):3133‐3144. 19075658 10.2174/092986708786848587

[40] Han S , Fink J , Jorg DJ , et al. Defining the identity and dynamics of adult gastric isthmus stem cells. Cell Stem Cell. 2019;25(3):342‐356.e347. 31422913 10.1016/j.stem.2019.07.008 PMC6739486

[41] McCracken KW , Cata EM , Crawford CM , et al. Modelling human development and disease in pluripotent stem‐cell‐derived gastric organoids. Nature. 2014;516(7531):400‐404. 25363776 10.1038/nature13863 PMC4270898

[42] Boccellato F , Woelffling S , Imai‐Matsushima A , et al. Polarised epithelial monolayers of the gastric mucosa reveal insights into mucosal homeostasis and defence against infection. Gut. 2019;68(3):400‐413. 29467166 10.1136/gutjnl-2017-314540 PMC6580761

[43] Morrison SJ , Spradling AC . Stem cells and niches: mechanisms that promote stem cell maintenance throughout life. Cell. 2008;132(4):598‐611. 18295578 10.1016/j.cell.2008.01.038 PMC4505728

[44] Huch M , Dorrell C , Boj SF , et al. In vitro expansion of single Lgr5(+) liver stem cells induced by Wnt‐driven regeneration. Nature. 2013;494(7436):247‐250. 23354049 10.1038/nature11826 PMC3634804

[45] Fujii M , Matano M , Toshimitsu K , et al. Human intestinal organoids maintain self‐renewal capacity and cellular diversity in niche‐inspired culture condition. Cell Stem Cell. 2018;23(6):787‐793.e6. 30526881 10.1016/j.stem.2018.11.016

[46] Colombo M , Raposo G , Thery C . Biogenesis, secretion, and intercellular interactions of exosomes and other extracellular vesicles. Annu Rev Cell Dev Biol. 2014;30:255‐289. 25288114 10.1146/annurev-cellbio-101512-122326

[47] Chen G , Huang AC , Zhang W , et al. Exosomal PD‐L1 contributes to immunosuppression and is associated with anti‐PD‐1 response. Nature. 2018;560(7718):382‐386. 30089911 10.1038/s41586-018-0392-8 PMC6095740

[48] Ong SG , Lee WH , Huang M , et al. Cross talk of combined gene and cell therapy in ischemic heart disease role of exosomal MicroRNA transfer. Circulation. 2014;130(11):S60. 25200057 10.1161/CIRCULATIONAHA.113.007917 PMC4862832

[49] Codispoti B , Marrelli M , Paduano F , Tatullo M . NANOmetric BIO‐banked MSC‐derived exosome (NANOBIOME) as a novel approach to regenerative medicine. J Clin Med. 2018;7(10):357. 10.3390/jcm7100357 PMC6210357 30326618

[50] Castrén M . Neural stem cells. Results Probl Cell Differ. 2012;54:33‐40. 22009346 10.1007/978-3-642-21649-7_3

[51] Khan M , Nickoloff E , Abramova T , et al. Embryonic stem cell‐derived exosomes promote endogenous repair mechanisms and enhance cardiac function following myocardial infarction. Circ Res. 2015;117(1):52‐64. 25904597 10.1161/CIRCRESAHA.117.305990 PMC4482130

[52] Park JH , Byeun DG , Choi JK . Progress, prospects, and limitations of organoid technology. Organoid. 2022;2:e9.

[53] Hughes CS , Postovit LM , Lajoie GA . Matrigel: a complex protein mixture required for optimal growth of cell culture. Proteomics. 2010;10(9):1886‐1890. 20162561 10.1002/pmic.200900758

[54] Tibbitt MW , Anseth KS . Hydrogels as extracellular matrix mimics for 3D cell culture. Biotechnol Bioeng. 2009;103(4):655‐663. 19472329 10.1002/bit.22361 PMC2997742

[55] Hutanu D , Frishberg MD , Guo L , Darie CC . Recent applications of polyethylene glycols (PEGs) and PEG derivatives. Mod Chem Appl. 2014;2:1‐6.

[56] Tatullo M , Genovese F , Aiello E , et al. Phosphorene is the new graphene in biomedical applications. Materials. 2019;12(14):2301. 10.3390/ma12142301 PMC6678593 31323844

[57] Tatullo M , Zavan B , Genovese F , et al. Borophene is a promising 2D allotropic material for biomedical devices. Appl Sci. 2019;9(17):3446.

[58] Thorne CA , Chen IW , Sanman LE , Cobb MH , Wu LF , Altschuler SJ . Enteroid monolayers reveal an autonomous WNT and BMP circuit controlling intestinal epithelial growth and organization. Dev Cell. 2018;44(5):624‐633.e624. 29503158 10.1016/j.devcel.2018.01.024 PMC5849535

