# Supplementary figures and images for: Establishment and characterization of adult human gastric epithelial progenitor‐like cell lines
Source: Cell Prolif. 2022 Nov 4;56(6):e13355. doi: 10.1111/cpr.13355 (PMC10280148; doi:10.1111/cpr.13355)

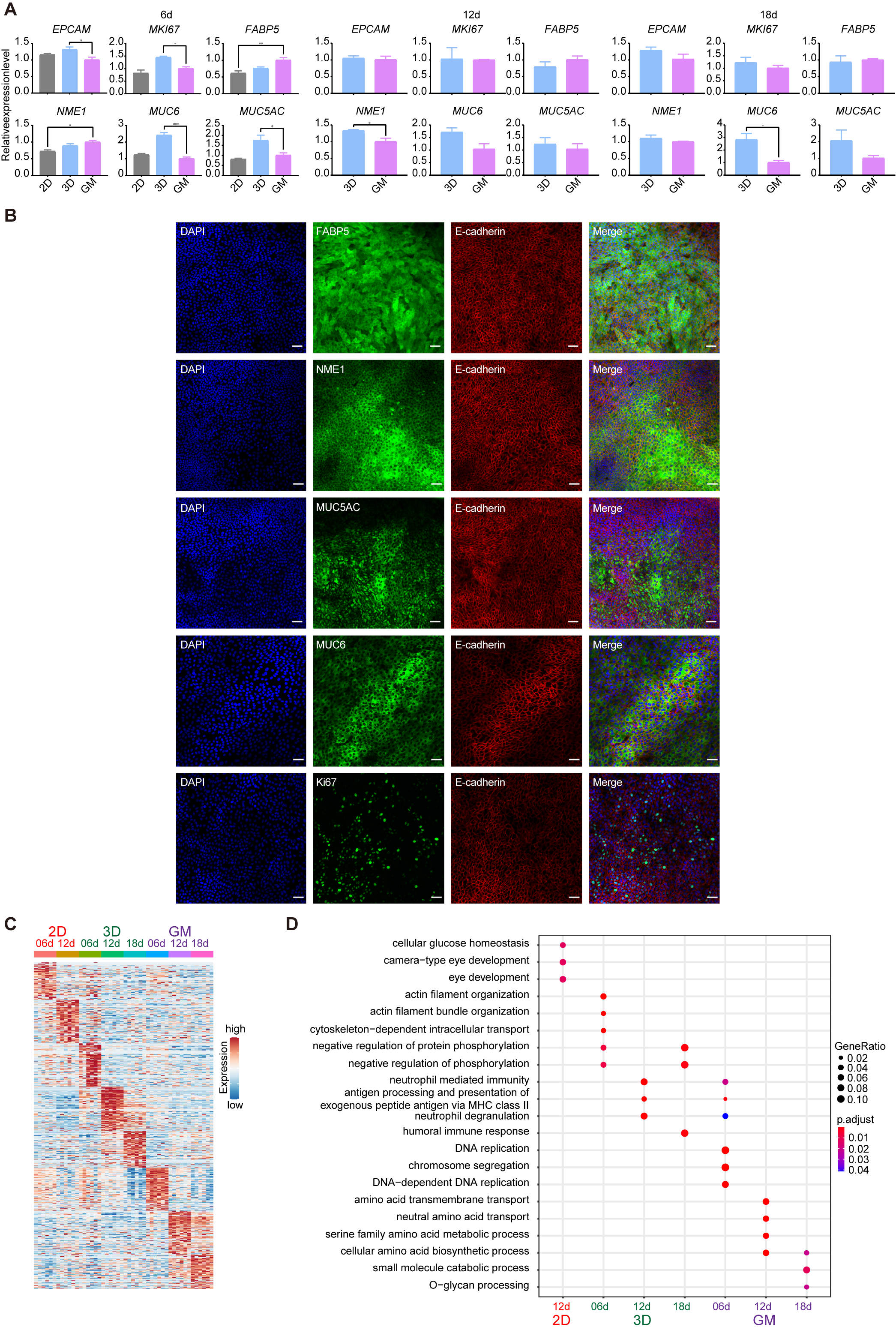

Supplement: Supplementary file 1 — FIGURE S1 Characterization of hGEPL cells [file CPR-56-e13355-s001.tif]

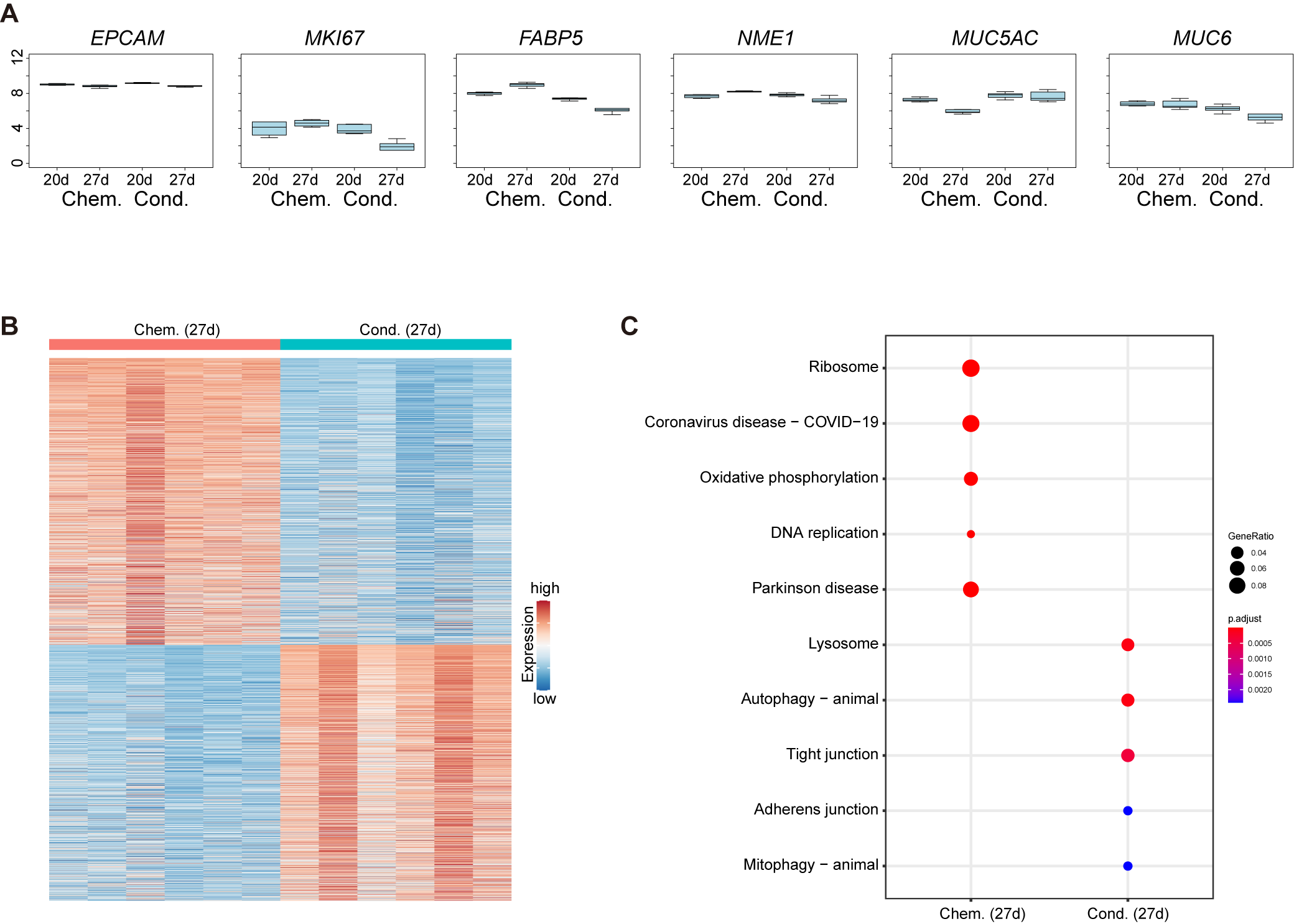

Supplement: Supplementary file 2 — FIGURE S2 Differences between hGEPL cells cultured in Chem. and Cond. media [file CPR-56-e13355-s002.tif]

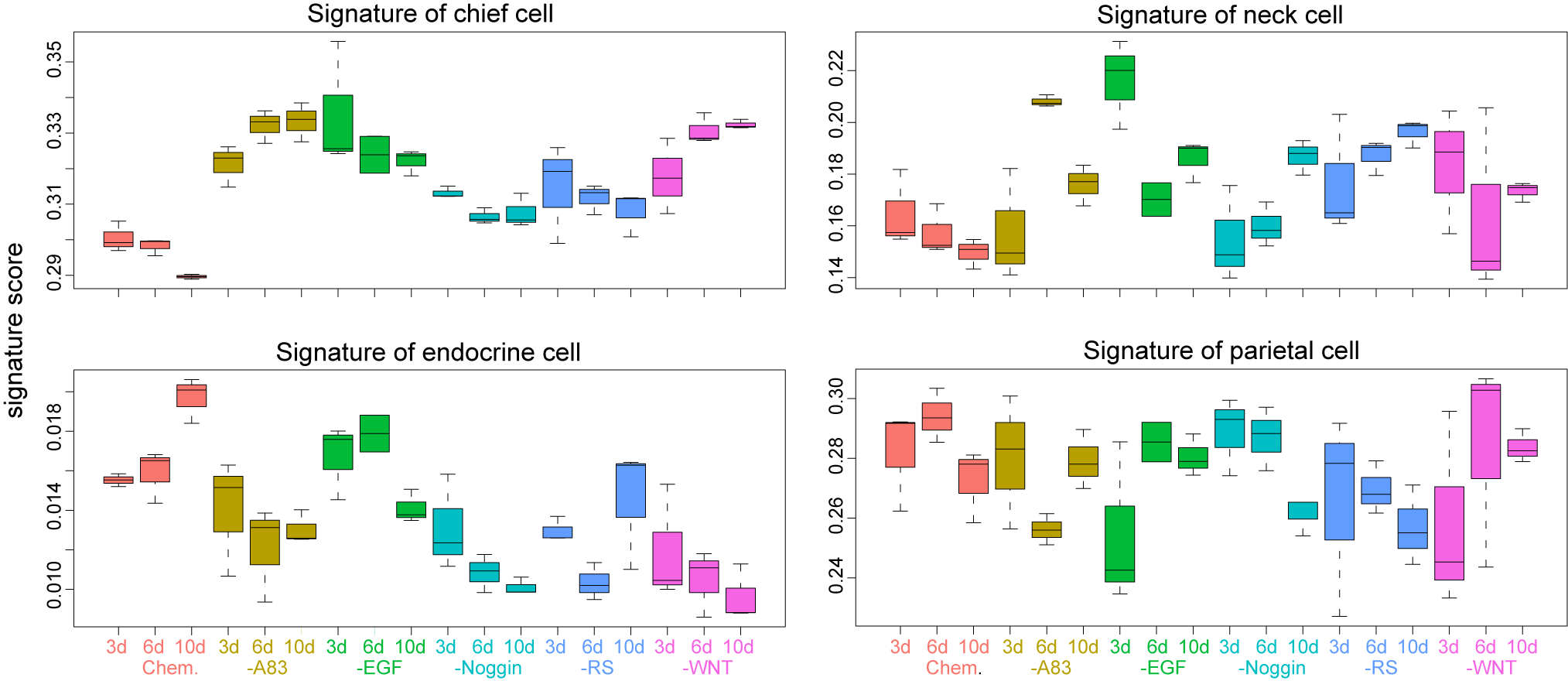

Supplement: Supplementary file 3 — FIGURE S3 The signature scores of in vivo gastric epithelial cell types in hGEPL cells after the removal of a certain growth factor [file CPR-56-e13355-s003.tif]
